# Early Post-Operative Pancreatitis and Systemic Inflammatory Response Assessed by Serum Lipase and IL-6 Predict Pancreatic Fistula

**DOI:** 10.1007/s00268-020-05768-9

**Published:** 2020-09-08

**Authors:** S. Gasteiger, F. Primavesi, G. Göbel, E. Braunwarth, B. Cardini, M. Maglione, S. Sopper, D. Öfner, S. Stättner

**Affiliations:** 1 Department of Visceral, Transplant and Thoracic Surgery, Medical University of Innsbruck, Anichstrasse 35, 6020 Innsbruck, Austria; 2 Department of Medical Statistics, Informatics and Health Economics, Medical University of Innsbruck, Schoepfstrasse 41, 6020 Innsbruck, Austria; 3 Department of Haematology and Oncology, Medical University of Innsbruck, Anichstrasse 35, 6020 Innsbruck, Austria; 4 Department of General, Vascular and Visceral Surgery, Salzkammergut Klinikum, Dr.-Wilhelm-Bock-Straße 1, 4840 Vöcklabruck, Austria

## Abstract

**Background:**

Post-operative pancreatic fistula (POPF) remains a critical complication after pancreatic resection. This prospective pilot study evaluates perioperative markers of pancreatitis and systemic inflammation to predict clinically relevant grade B/C-POPF (CR-POPF).

**Methods:**

All patients undergoing pancreatic resection from December 2017 to April 2019 were prospectively enrolled. Surgical procedures and outcomes were correlated with perioperative blood markers. ROC analysis was performed to assess their predictive value for CR-POPF. Cut-offs were calculated with the Youden index.

**Results:**

In total, 70 patients were analysed (43 pancreatoduodenectomies and 27 distal pancreatectomies). In-hospital/90-d mortality and morbidity were 5.7/7.1% (*n* = 4/*n* = 5) and 75.7% (*n* = 53). Major complications (Clavien–Dindo ≥ 3a) occurred in 28 (40.0%) patients, CR-POPF in 20 (28.6%) patients. Serum lipase (cut-off > 51U/L) and IL-6 (> 56.5 ng/l) on POD3 were significant predictors for CR-POPF (AUC = 0.799, 95%-CI 0.686–0.912 and AUC = 0.784, 95%-CI 0.668–0.900; combined AUC = 0.858, 95%-CI 0.758–0.958; all *p* < 0.001). Patients with both or one factor(s) above cut-off more frequently developed CR-POPF than cases without (100 vs. 50% vs. 7.5%, *p* < 0.001). This also applied for overall and severe complications (*p* = 0.013 and *p* = 0.009).

**Conclusions:**

Post-operative pancreatitis and inflammatory response are major determinants for development of POPF. A combination of serum lipase and IL-6 on POD3 is a highly significant early predictor of CR-POPF and overall complications, potentially guiding patient management.

**Clinical trial registration:**

The study protocol was registered at clinicaltrials.gov (NCT04294797)

**Electronic supplementary material:**

The online version of this article (10.1007/s00268-020-05768-9) contains supplementary material, which is available to authorized users.

## Introduction

Pancreatic resections (PR) represent surgical procedures with considerable rates of mortality and morbidity. Due to constant improvements in surgical technique and complication management, post-operative death has significantly decreased over the last decades currently ranging around 5% in most centres [1–3]. In contrast, morbidity following PR remains frequent and gradually increases with the complexity of the procedure performed [4]. While enhanced recovery concepts have resulted in a decline in general post-operative morbidity such as pneumonia or wound infections, specific complications like post-operative pancreatic fistula (POPF), postpancreatectomy haemorrhage (PPH) and delayed gastric emptying (DGE) remain common and often trigger other complications [5].

Early anticipation and treatment of clinically relevant grade B or C POPF (CR-POPF) is of utmost importance to prevent fatal outcome. While most pancreatic fistula is self-limiting without the need of intervention (biochemical leak), persistent uncontrolled and insufficiently drained pancreatic juice leakage can lead to a series of disastrous events including destruction of surrounding tissue and erosion of blood vessels resulting in life-threatening infections, sepsis and haemorrhage [6]. In cancer patients, such major complications may delay the start of adjuvant chemotherapies and influence the patient’s prognosis [7, 8]. In order to aid with timely detection of potentially severe CR-POPF, evaluation of predictive biomarkers that could be able to differentiate early between non-significant biochemical leaks and CR-POPF is of urgent interest.

The aim of this prospective study was to assess readily available biomarkers of local pancreatic inflammation and systemic inflammatory response in regard to their association with development of CR-POPF, 90-day morbidity and mortality following PR as a tool for post-operative decision-making.

## Methods

Following approval by the local ethics committee (study number 1081/2017), all patients undergoing pancreatic head resection or distal pancreatectomy at the Department of Visceral, Transplant and Thoracic Surgery, Medical University of Innsbruck, Austria, between December 2017 and May 2019 were enrolled in this prospective study, and written informed consent was obtained. Exclusion criteria comprised total pancreatectomy, duodenum preserving pancreatic head resection, enucleation and cases with unresectability after exploration. The study was conducted in accordance with the Helsinki declaration 2013 and the STROBE checklist [9], and the protocol was registered at clinicaltrials.gov (NCT04294797).

POPF was defined according to the 2016 update of the International Study Group for Pancreatic Surgery (ISGPS), [10] postpancreatectomy haemorrhage (PPH) and delayed gastric emptying (DGE) according to the respective 2007 ISGPS definitions [11, 12]. Post-operative pancreatitis (POAP) was defined by Connor’s proposal, with serum amylase/lipase values being increased above the upper limit of normal (53 and 60 U/L, respectively, according to our local laboratory) between surgery (skin closure) and end of POD1 [13]. Failure to rescue (FTR) was the rate of deaths in the total number of patients experiencing complications. All complications were assessed within 90 days after surgery, graded according to the Clavien–Dindo classification [14] and recorded prospectively through our surgical units’ auditable database (ChiBase).

Preoperatively, routine laboratory parameters were assessed on the day before surgery (white blood cell count, amylase, lipase, albumin, C-reactive protein, IL-6, IL-8, procalcitonin, TNF-alpha). The same markers were evaluated on the morning of POD1 and POD3 with additional measurement of drain fluid amylase and lipase levels.

### Statistical analysis

Data are reported as mean (SD), median (range) or numbers with percentages. ROC analysis was performed to assess laboratory values regarding prediction of CR-POPF, and optimal cut-offs were calculated with the Youden index. Risk groups were compared regarding outcome parameters with appropriate two-tailed contingency tests for categorical variables and with the Kruskal–Wallis test for continuous data with non-normal distribution. *P* values ≤ 0.05 were considered statistically significant, and analyses were performed using SPSS® version 23 (IBM, Armonk, New York, USA). Perioperative dynamics of serum markers were displayed with GraphPad Prism 8.1.2 (GraphPad Software Inc., La Jolla, California, USA).

## Results

### Patient characteristics and surgical procedure

A total of 70 patients were analysed, of which 43 (61.4%) underwent pancreatic head resection and 27 (38.6%) distal pancreatectomy. Their baseline data are summarized in Table 1. The indication for resection was malignancy in 56 patients (80.0%) with the majority (*n* = 36/51.4%) suffering from pancreatic ductal adenocarcinomas (PDACs). Pancreatic anastomosis was performed according to Blumgart’s technique [15] in 76.7% of patients and in Neuhaus technique in 23.3% [16]. Among head resections, 12 patients (27.9%) underwent preoperative biliary drainage (PBD) and 15 cases (34.9%) vascular resection (mostly portal vein or superior mesenteric vein). In the whole cohort, 9 patients (12.9%) received additional minor hepatectomies or gastric resections.

**Table 1:** Patient characteristics and surgical details (*n* = 71)

Characteristics	*n* (%)
Age (years)	
< 60	29 (41.4)
60–69	18 (25.7)
70–79	16 (22.9)
≥ 80	7 (10.0)
Gender	
Male	38 (54.3)
Female	32 (45.7)
BMI*	25.1 (16.1–43)
Chronic pancreatitis	8 (11.4)
Pre-existing diabetes mellitus	12 (17.1)
Surgical procedure	
PPPD	42 (60.0)
PRPD	1 (1.4)
Laparoscopic distal pancreatectomy	18 (25.7)
Open distal pancreatectomy	9 (12.9)
Indication	
PDAC	36 (51.4)
pNET	12 (17.1)
dCCC	4 (5.7)
IPMN	4 (5.7)
PanIN	1 (1.4)
Chronic pancreatitis	3 (4.3)
Metastases from RCC	1 (1.4)
Other	9 (12.9)

*BMI* body mass index; *PPPD* pylorus-preserving pancreatoduodenectomy; *PRPD* pylorus-resecting pancreatoduodenectomy; *PDAC* pancreatic ductal adenocarcinoma; *NET* neuroendocrine tumour; *dCCC* distal cholangiocellular carcinoma; *IPMN* intraductal papillary mucinous neoplasm; *PanIN* intraepithelial neoplasm; *RCC* renal cell carcinoma

*Values are median (range)

### Perioperative mortality and morbidity

In-hospital mortality was 5.7% (*n* = 4), and 90-day mortality and morbidity were 7.1% (*n* = 5) and 75.7% (*n* = 53), respectively. This included meticulous documentation of minor grade 1 or 2 complications (Table 2). Major complications (≥3a) occurred in 28 patients (40.0%). History of PBD was not significantly associated with major complications or CR-POPF (41.7% vs. 45.2% and 8.3% vs. 29.0%; *p* = 0.836 and *p* = 0.237). The FTR rate was 9.3%. Cause of in-hospital mortality in one patient was multiorgan failure due to gastric ischemia after distal pancreatectomy with partial gastric resection. Another patient developed multiorgan failure following concurrent insufficiency of the pancreatojejunostomy and hepaticojejunostomy, and two patients died due to cardiac infarction. One patient died on POD80 due to progressive renal insufficiency and ascites after initial discharge (suspected portal vein thrombosis). Biochemical leak was recorded in 16 patients (22.9%) and CR-POPF in 20 patients (28.6%) including 13 grade B (18.6%) and 7 grade C fistula (10.0%), respectively. PPH occurred in 7 patients (10.0%), DGE in 8 patients (11.4%) and POAP in 25 patients (35.7%). As shown in Table 2, although the overall morbidity and the rate of POPF including biochemical leak were significantly higher in distal resections, 90-day mortality was lower (3.7%) than after pancreatoduodenectomy (9.3%) (*p* = 0.642). All but one death occurred in vascular resection patients.

**Table 2:** 90-day morbidity and mortality

	Total	Pancreatic head resections	Distal pancreatectomies	*P* value
	70 (100%)	43 (100%)	27 (100%)	
Overall mortality	5 (7.1)	4 (9.3)	1 (3.7)	0.642
In-hospital mortality	4 (5.7)	3 (6.9)	1 (3.7)	0.566
Overall morbidity	53 (75.7)	29 (67.4)	24 (88.9)	0.049
Clavien–Dindo 1	14 (20.0)	5 (11.6)	9 (33.3)	0.035
Clavien–Dindo 2	11 (15.7)	5 (11.6)	6 (22.2)	0.315
Clavien–Dindo 3a	6 (8.6)	3 (6.9)	3 (11.1)	0.670
Clavien–Dindo 3b	12 (17.1)	7 (16.3)	5 (18.5)	0.809
Clavien–Dindo 4a	4 (5.7)	4 (9.3)	0	0.154
Clavien–Dindo 4b	1 (1.4)	1 (2.3)	0	0.425
Overall POPF	36 (51.4)	14 (32.6)	22 (81.5)	< 0.001
Biochemical leak	16 (22.9)	4 (9.3)	12 (44.4)	0.001
Grade B	13 (18.6)	5 (11.6)	8 (29.6)	0.112
Grade C	7 (10.0)	5 (11.6)	2 (7.4)	0.699
POAP	25 (35.7)	9 (20.9)	16 (59.3)	0.002
PPH	7 (10.0)	5 (11.6)	2 (7.4)	0.699
DGE	8 (11.4)	8 (18.6)	0	0.020

*POPF* post-operative pancreatic fistula; *POAP* post-operative acute pancreatitis; *PPH* post-operative pancreatic haemorrhage; *DGE* delayed gastric emptying

### Clinical factors associated with POPF

CR-POPF rates did not differ significantly between distal pancreatectomies and pancreatic head resections (*n* = 10/37.0% vs. *n* = 10/23.3%, *p* = 0.279). Also, the incidence of CR-POPF was not different in regard to surgical technique of pancreatic anastomosis (Blumgart vs. Neuhaus; *n* = 6/33 (18.2%) vs. *n* = 4/10 (40%); *p* = 0.206). The presence of POAP versus no POAP was a strong predictor of POPF including biochemical leak (84.0% vs. 33.3%, *p* < 0.001), although it was not significantly associated with CR-POPF only (36.0% vs. 24.4%, *p* = 0.409). Moreover, POAP occurred more often following distal pancreatectomy compared to head resection (59.3% vs. 20.9%, *p* = 0.002).

### Laboratory markers associated with CR-POPF

In our cohort, 24 patients fulfilled the established ISGLS criteria of POPF/biochemical leak on POD3 (drain amylase > 3 × upper normal limit of serum amylase) [10]. Subsequently, only 11 (45.8%) of these patients eventually developed CR-POPF (sensitivity 55.0%, specificity 71.1%, negative-predictive value (NPV) 78.1%; AUC 0.720, 95%-CI 0.594–0.845). Although individual drain amylase levels on POD3 predicted CR-POPF in ROC analysis (*p* = 0.004), correlation of the predefined drain amylase cut-off on POD3 according to ISGLS (>159 U/L in our department) did just not reach statistically significance (*p* = 0.055).

**Figure 1:**
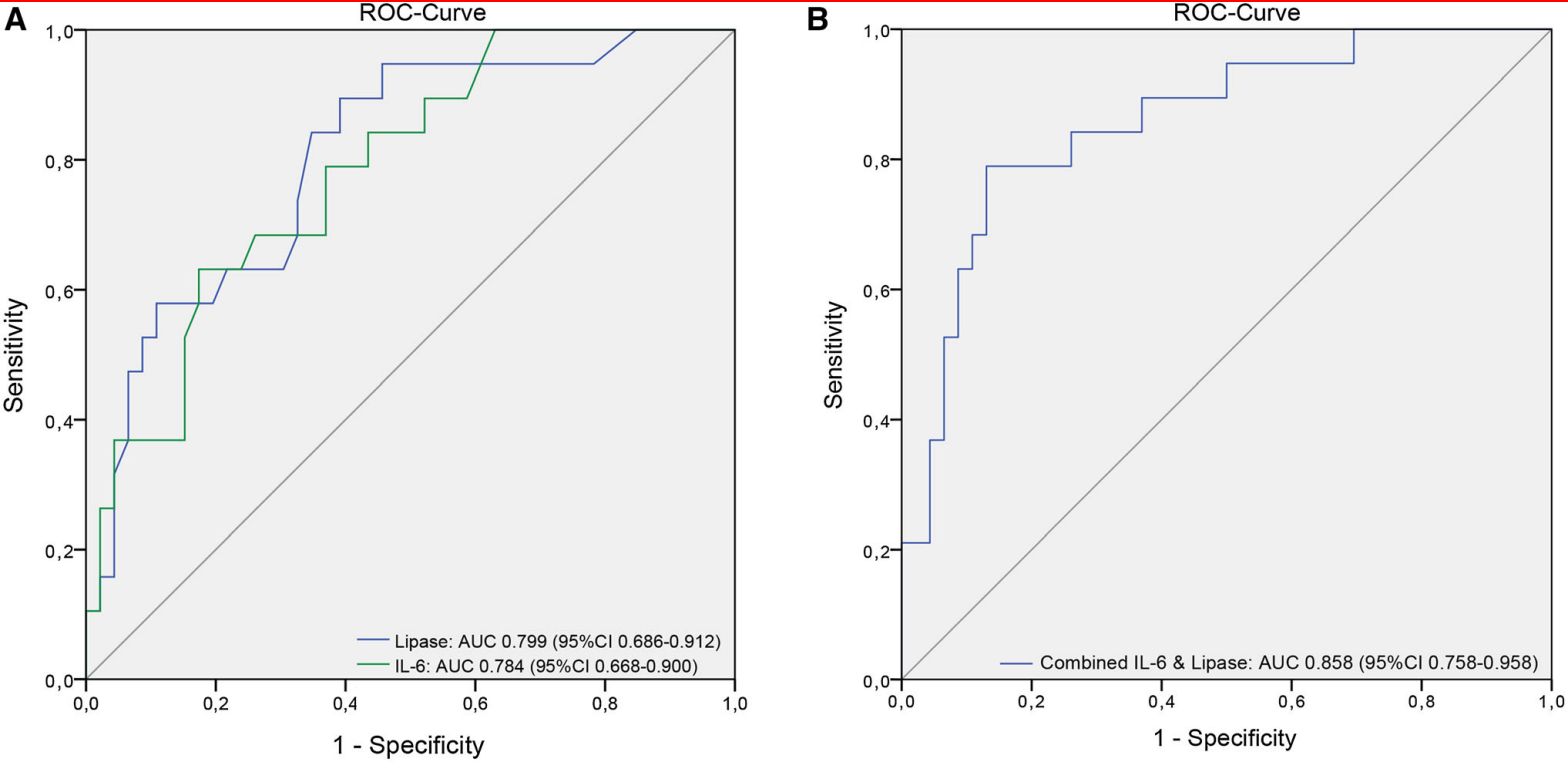
ROC analysis of the predictive value of lipase and IL-6 on POD3 (**a**) and both factors combined (**b**) for development of clinically relevant post-operative pancreatic fistula (CR-POPF)

**Figure 2:**
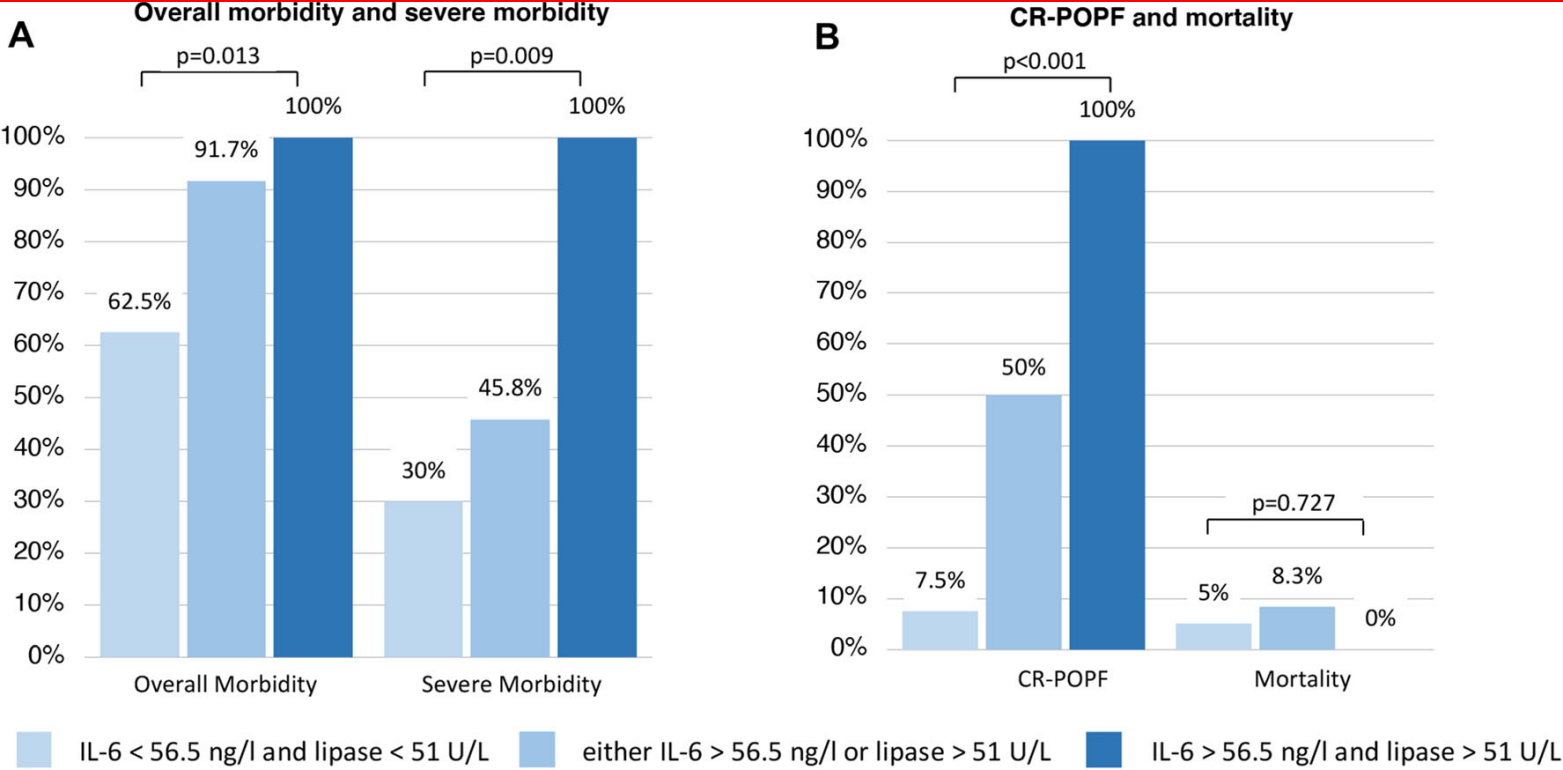
Post-operative overall and severe morbidity (**a**) and clinically relevant post-operative pancreatic fistula (CR-POPF) as well as mortality (**b**) stratified by the presence of none, one or both serum markers (lipase and IL-6) above the calculated cut-off

**Figure 3:**
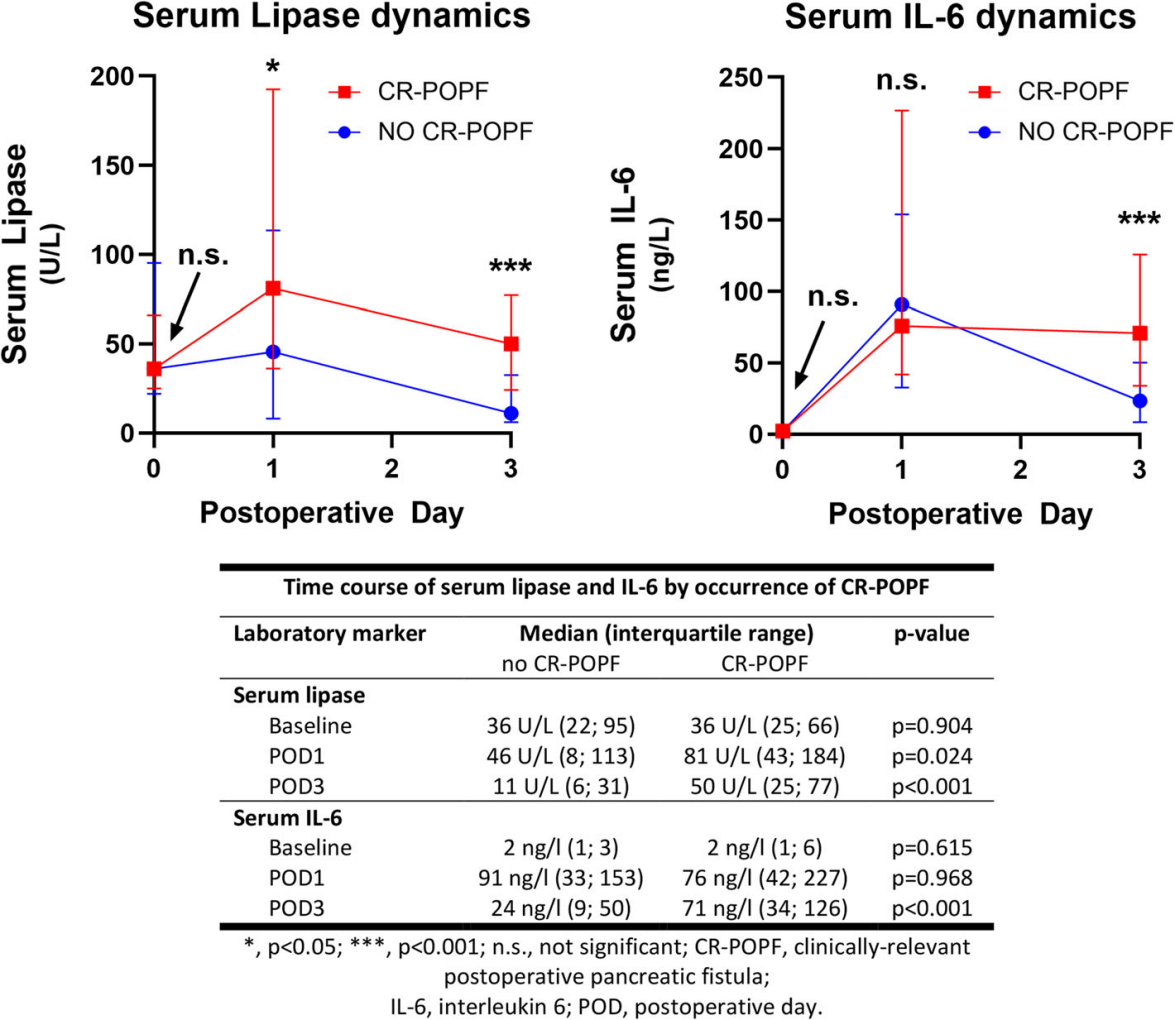
Time courses of serum lipase and IL6 according to patients stratified by further development of clinically relevant post-operative pancreatic fistula (CR-POPF). XY-line graphs are shown with median and interquartile range error bars

Table 3 shows ROC analysis of perioperative biomarkers and their predictive value for development of CR-POPF. Two markers related to local or systemic inflammation showing the highest ROC-AUC were selected for further cut-off analysis. Serum lipase (cut-off ≥ 51 U/L; sensitivity 50.0%, specificity 91.8%, NPV 81.8%; AUC 0.799, 95%-CI 0.686–0.912) and IL-6 (cut-off ≥ 56.5 ng/l; sensitivity 63.2%, specificity 82.6%, NPV 84.4%; AUC 0.784, 95%-CI 0.668–0.900) on POD3 showed the strongest association with CR-POPF: 10 of 14 patients (71.4%) with high serum lipase compared to 10 of 55 patients (18.2%) with low lipase and 12 of 20 patients (60%) with high IL-6 compared to 7 of 45 patients (15.6%) with low IL-6 developed CR-POPF (both *p* = 0.001). Combining both markers by multivariable logistic regression resulted in further improved predictive power (AUC 0.858, 95%-CI 0.758–0.958; Fig. 1). Patients with none of those factors above cut-off had a comparably low rate (7.5%) of developing a CR-POPF in the further post-operative course (NPV 92.5%, 95%-CI 80.1–97.4; sensitivity 85%, specificity 75.5%). Intriguingly, the presence of one or both markers raised was not only associated with a stepwise increase in CR-POPF (50% and 100%; *p* < 0.001) but also in overall and severe morbidity (*p* = 0.013 and *p* = 0.009; Fig. 2). Accordingly, we observed a significant steady increase in median LOS (Median 10 vs. 15 vs. 25 days; *p* = 0.017). However, there was no clear association with post-operative mortality (*p* = 0.727). Perioperative time courses of serum lipase and IL-6 stratified by further development of CR-POPF are shown in Fig. 3.

**Table 3:** Different perioperative laboratory markers and their predictive value for development of CR-POPF

Laboratory marker	Total, *n* = 70	
	AUC-ROC	*P*
Preoperative baseline		
Serum amylase	0.528 (95%CI 0.389–0.667)	0.722
Serum lipase	0.509 (95%CI 0.365–0.654)	0.904
Albumin	0.543 (95%CI 0.398–0.687)	0.584
CRP	0.472 (95%CI 0.325–0.618)	0.711
IL-6	0.539 (95%CI 0.372–0.706)	0.615
IL-8	0.420 (95%CI 0.280–0.560)	0.302
Procalcitonin	0.397 (95%CI 0.246–0.547)	0.200
TNF-alpha	0.494 (95%CI 0.336–0.653)	0.940
Leucocytes	0.620 (95%CI 0.475–0.765)	0.119
POD 1		
Drain amylase	0.694 (95%CI 0.554–0.835)	0.016
Serum amylase	0.638 (95%CI 0.501–0.775)	0.075
Serum lipase	0.675 (95%CI 0.537–0.813)	0.024
Albumin	0.549 (95%CI 0.405–0.692)	0.526
CRP	0.514 (95%CI 0.355–0.673)	0.856
IL-6	0.503 (95%CI 0.344–0.663)	0.968
IL-8	0.524 (95%CI 0.374–0.674)	0.757
Procalcitonin	0.477 (95%CI 0.305–0.650)	0.773
TNF-alpha	0.513 (95%CI 0.368–0.659)	0.867
Leucocytes	0.612 (95%CI 0.481–0.743)	0.145
POD 3		
Drain amylase	0.728 (95%CI 0.592–0.863)	0.004
Serum amylase	0.720 (95%CI 0.594–0.845)	0.004
Serum lipase	0.799 (95%CI 0.686–0.912)	<0.001
Albumin	0.411 (95%CI 0.248–0.574)	0.272
CRP	0.664 (95%CI 0.528–0.800)	0.033
IL-6	0.784 (95%CI 0.668–0.900)	<0.001
IL-8	0.764 (95%CI 0.647–0.881)	0.001
Procalcitonin	0.531 (95%CI 0.353.0.709)	0.704
TNF-alpha	0.612 (95%CI 0.468–0.756)	0.158
Leucocytes	0.626 (95%CI 0.472–0.780)	0.099

*CRP* C-reactive protein; *CR-POPF* clinically relevant post-operative pancreatic fistula; IL-6 interleukin 6; IL-8 interleukin 8; *POD* post-operative day; *TNF* tumour necrosis factor alpha;

Perioperative IL-6 and lipase levels did not differ among patients with PBD and without (IL-6 preoperative 2.45 vs. 2.25 ng/l (*p* = 0.330), POD3 32.0 vs. 25.5 ng/l (*p* = 0.913); lipase preoperative 44U/L vs. 36U/L (*p* = 0.481), POD3 8.5 vs. 11.0U/L (*p* = 0.303)).

### Subgroup analysis of pancreatic head and distal resections

The combination of IL-6 and lipase showed a strong correlation with CR-POPF independently of the performed procedure with an AUC of 0.880 (95%-CI 0.754–1.000) following pancreatic head resections (*p* < 0.001) and an AUC of 0.826 (95%-CI 0.641–1.000) following distal pancreatectomies (*p* = 0.008). However, in distal resections, IL-8 in particular showed a higher AUC than IL-6. Supplemental Table 1 portrays ROC subgroup analysis for all markers.

## Discussion

The present study prospectively assessed perioperative biomarkers and their association with complications including POPF after PR. It establishes a clear link between markers of local pancreatic inflammation (serum lipase), measurable systemic response (IL-6) and risk of development of CR-POPF. These markers are detectable early post-operatively, clinically well established and most importantly predict CR-POPF as well as overall morbidity with significant accuracy in both pancreatoduodenectomy and distal resections, although with higher diagnostic yield in pancreatoduodenectomies.

First, we have confirmed recent findings from others, showing that PR remain procedures associated with significant post-operative morbidity and mortality even in high-volume units. Four patients died during hospital stay with two of them due to surgical complications and two due to cardiac infarction after an initially uneventful post-operative course. To prevent future cardiac complications in our institution, preoperative work-up for major abdominal procedures now includes routine cardiac evaluation with cardio-pulmonary exercise testing and—if indicated—coronary artery angiography. Surgical complication related in-hospital mortality in this series was 2.8%, which is in line with that of other high-volume centres [1, 2] and comparable to previously published data of our centre [3]. The high number of vascular reconstructions and concurrent other organ resections shows the progressive approach recently applied in our institution, potentially suggesting a need for optimization in patient selection as well as perioperative management. Overall complication rate in our series reached 75.7%, which also seems high compared to retrospective series with complication rates between 30 and 60% [17]. Since 35% of our post-operative complications are classified as Clavien–Dindo 1–2 with no relevant impact on the further post-operative course, this observed difference is most certainly a result of meticulous prospective auditable documentation. Major complications occurred in 40% of patients, which is comparable to results reported by other prospective series or randomized trials [18–20]. Rates of pancreatic fistula highly differ in the literature ranging from 2–63% [6, 21, 22]. In our cohort, 28.6% of patients experienced CR-POPF. Early recognition of patients potentially developing severe fistula allows a personalized approach in post-operative management. While high-risk cases might benefit from early initiation of diagnostic and therapeutic steps, patients at low risk for CR-POPF can be allocated to early oral feeding pathways and undergo timely removal of their perianastomotic drains [23].

The post-operative systemic response to a local inflammatory stimulus is strongly related to complications after gastrointestinal surgery [24]. Van Hilst et al. showed higher IL-6 levels in patients with major complications and CR-POPF in 38 patients in the LEOPARD-2 trial [20, 25]. IL-6 is a cytokine that induces the production of acute-phase proteins such as CRP in the liver. IL-6 levels at 24 h post-operatively in abdominal surgery have been previously shown as accurate in predicting complications as CRP at 72 h [20, 24, 25]. In our analysis, IL-6 at POD3 was superior to IL-6 at POD1 and CRP at any time point in terms of accuracy to predict CR-POPF (Table 3).

Furthermore, the measurable response to pancreatitis (serum amylase and lipase) has previously been assessed for the prediction of CR-POPF [26, 27]. While both markers on POD3 were strongly associated with CR-POPF in our cohort, their predictive value on POD1 was of borderline significance. The presence of POAP as defined by Connor [13] was only associated with further development of POPF when biochemical (not clinically relevant) leaks were included. This suggests a limited power in our cohort with a considerably low number of patients developing POAP (35.7%) compared to 55.8% in a previous Italian study involving 292 patients [28]. In another retrospective analysis, serum lipase at POD1 was assessed in 98 patients undergoing pancreatoduodenectomy, reporting that patients with levels below a cut-off of 44.5 U/L had a substantially lower probability to develop CR-POPF [29]. Intriguingly, our calculated cut-off of 51 U/L was quite comparable, despite measurement on POD3.

Attempting to incorporate both the local pancreatic remnant inflammatory state and the systemic response to improve early post-operative risk stratification, we combined serum lipase and IL-6 on POD3. This ultimately resulted in three groups of patients with significantly different risks for the development of CR-POPF. While all patients with both markers high developed CR-POPF, the rate was only 7.5% with no factor raised above cut-off. The resulting high NPV (92.5%) was markedly superior to that of drain amylase (78.1%). This could strongly impact clinical management, enhanced recovery programs and drainage removal strategies [30]. All patients with both markers increased should be observed with great vigilance to timely initiate further diagnostics and therapeutics and prevent fatal complications of insufficiently drained and persistent fistulas. Importantly, although risk factors for CR-POPF differ between pancreatic head and distal resections, the correlation of IL-6 and serum lipase with later CR-POPF was highly predictive in both subgroups.

Limitations of our study include the single-centre design, inclusion of head and distal resections, application of different techniques for pancreatojejunostomy anastomosis, pooling of open and laparoscopic procedures and a rather conservative drain management. The cohort consisted of a relevant number of patients < 60 years (>40%) and with chronic pancreatitis (>10%), which needs to be taken into consideration regarding external validity. Further prospective, international validation with a larger sample size and detailed subgroup analysis should be performed. Particularly for pancreatoduodenectomies, our score should be compared to others readily available such as the Fistula Risk Score [31]. Moreover, its applicability in differing perioperative strategies (anastomotic reconstruction techniques, drain management) requires validation.

In conclusion, post-operative complications and POPF remain a major issue even in high-volume centres [21]. The local pancreatic and systemic inflammatory response appears to be decisive, showing a strong association of POD3 serum IL-6 and lipase with CR-POPF and severe complications. The resulting risk groups according to proposed marker cut-off levels allow for improved stratification compared to established criteria such as drain amylase. In case of confirmative validation, these results might foster the development of a new approach in predicting and grading CR-POPF.

## Electronic supplementary material

Below is the link to the electronic supplementary material.

Supplementary file1 (DOCX 17 kb)

## Abbreviations

BMI: Body mass index
dCCC: Distal cholangiocellular carcinoma
CR-POPF: Clinically relevant post-operative pancreatic fistula
DGE: Delayed gastric emptying
DM: Diabetes mellitus
FTR: Failure to rescue
IPMN: Intraductal papillary mucinous neoplasm
NET: Neuroendocrine tumour
NPV: Negative-predictive value
ISGPS: International study group for pancreatic surgery
PanIN: Pancreatic intraepithelial neoplasia
PBD: Preoperative biliary drainage
PDAC: Pancreatic ductal adenocarcinoma
POD: Post-operative day
POPF: Post-operative pancreatic fistula
POAP: Post-operative acute pancreatitis
PPH: Post-operative pancreatic haemorrhage
PPPD: Pylorus-preserving pancreatic head resection
PRPD: Pylorus-resecting pancreatic head resection
PR: Pancreatic resection
