# Supplementary material for: Early Post-Operative Pancreatitis and Systemic Inflammatory Response Assessed by Serum Lipase and IL-6 Predict Pancreatic Fistula
Source: World J Surg. 2020 Sep 8;44(12):4236–44. doi: 10.1007/s00268-020-05768-9 (PMC7599180; doi:10.1007/s00268-020-05768-9)
Supplement: Supplementary file 1 — Supplementary file1 (DOCX 17 kb) [file 268_2020_5768_MOESM1_ESM.docx]

| **Supplemental Table 1: Subgroup analysis of perioperative laboratory markers and their predictive value for development of CR-POPF in pancreatic head and distal resections.** | | | | |
| --- | --- | --- | --- | --- |
|  | Pancreatic head resections, n=43 | | Distal pancreatectomies, n=27 | |
| **Laboratory marker** | **AUC-ROC** | **P** | **AUC-ROC** | **P** |
| **Preoperative baseline** |  |  |  |  |
| Serum amylase | 0.536 (95%CI 0.350-0.723) | 0.730 | 0.493 (95%CI 0.267-0.720) | 0.957 |
| Serum lipase | 0.488 (95%CI 0.292-0.684) | 0.908 | 0.556 (95%CI 0.327-0.784) | 0.647 |
| Albumin | 0.497 (95%CI 0.291-0.703) | 0.976 | 0.547 (95%CI 0.316-0.778) | 0.698 |
| CRP | 0.391 (95%CI 0.186-0.596) | 0.301 | 0.624 (95%CI 0.390-0.857) | 0.292 |
| IL-6 | 0.544 (95%CI 0.337-0.750) | 0.679 | 0.547 (95%CI 0.283-0.811) | 0.688 |
| IL-8 | 0.453 (95%CI 0.276-0.630) | 0.658 | 0.412 (95%CI 0.186-0.638) | 0.451 |
| Procalcitonin | 0.392 (95%CI 0.169-0.616) | 0.331 | 0.493 (95%CI 0.253-0.733) | 0.955 |
| TNF-alpha | 0.522 (95%CI 0.304-0.740) | 0.836 | 0.536 (95%CI 0.301-0.771) | 0.767 |
| Leucocytes | 0.555 (95%CI 0.369-0.740) | 0.605 | 0.703 (95%CI 0.473-0.933) | 0.083 |
| **POD 1** |  |  |  |  |
| Drain amylase | 0.694 (95%CI 0.505-0,882) | 0.069 | 0.545 (95%CI 0.280-0.811) | 0.732 |
| Serum amylase | 0.729 (95%CI 0.549-0,909) | 0.031 | 0.435 (95%CI 0.200-0.671) | 0.581 |
| Serum lipase | 0.795 (95%CI 0.622-0.968) | 0.005 | 0.388 (95%CI 0.163-0.613) | 0.340 |
| Albumin | 0.579 (95%CI 0.380-0.778) | 0.455 | 0.419 (95%CI 0.195-0.643) | 0.493 |
| CRP | 0.621 (95%CI 0.397-0.845) | 0.250 | 0.518 (95%CI 0.293-0.743) | 0.880 |
| IL-6 | 0.626 (95%CI 0.442-0.811) | 0.250 | 0.394 (95%CI 0.148-0.640) | 0.366 |
| IL-8 | 0.569 (95%CI 0.369-0.769) | 0.530 | 0.547 (95%CI 0.319-0.775) | 0.688 |
| Procalcitonin | 0.633 (95%CI 0.420-0.846) | 0.206 | 0.373 (95%CI 0.127-0.618) | 0.293 |
| TNF-alpha | 0.611 (95%CI 0.424-0.799) | 0.312 | 0.491 (95%CI 0.273-0.710) | 0.940 |
| Leucocytes | 0.661 (95%CI 0.503-0.818) | 0.128 | 0.479 (95%CI 0.252-0.707) | 0.861 |
| **POD 3** |  |  |  |  |
| Drain amylase | 0.773 (95%CI 0.591-0.956) | 0.010 | 0.515 (95%CI 0.256-0.775) | 0.901 |
| Serum amylase | 0.771 (95%CI 0.606-0.937) | 0.010 | 0.641 (95%CI 0.423-0.858) | 0.236 |
| **Serum lipase** | **0.818 (95%CI 0.666-0.970)** | **0.003** | **0.784 (95%CI 0.605-0.964)** | **0.016** |
| Albumin | 0.371 (95%CI 0.188-0.609) | 0.266 | 0.373 (95%CI 0.147-0.600) | 0.292 |
| CRP | 0.779 (95%CI 0.630-0.927) | 0.008 | 0.500 (95%CI 0.259-0.741) | 1.000 |
| **IL-6** | **0.817 (95%CI 0.681-0.953)** | **0.003** | **0.740 (95%CI 0.527-0.952)** | **0.051** |
| IL-8 | 0.770 (95%CI 0.616-0.924) | 0.011 | 0.832 (95%CI 0.660-1.000) | 0.009 |
| Procalcitonin | 0.713 (95%CI 0.500-0.926) | 0.055 | 0.437 (95%CI 0.160-0.714) | 0.612 |
| TNF-alpha | 0.637 (95%CI 0.441-0.833) | 0.200 | 0.594 (95%CI 0.369-0.818) | 0.445 |
| Leucocytes | 0.542 (95%CI 0.308-0.777) | 0.687 | 0.668 (95%CI 0.455-0.881) | 0.152 |
| CRP, c-reactive protein; CR-POPF, clinically relevant postoperative pancreatic fistula; IL-6, interleukin 6; IL-8, interleukin 8; POD, postoperative day; TNF, tumor-necrosis-factor alpha; | | | | |
